# Oxytetracycline induces DNA damage and epigenetic changes: a possible risk for human and animal health?

**DOI:** 10.7717/peerj.3236

**Published:** 2017-04-27

**Authors:** Adriana Gallo, Rosaria Landi, Valentina Rubino, Alessandro Di Cerbo, Angela Giovazzino, Anna Teresa Palatucci, Sara Centenaro, Gianandrea Guidetti, Sergio Canello, Laura Cortese, Giuseppina Ruggiero, Andrea Alessandrini, Giuseppe Terrazzano

**Affiliations:** 1 Institute of Experimental Endocrinology and Oncology (IEOS), National Research Council (CNR), Naples, Italy; 2 Department of Molecular Medicine and Medical Biotechnology, University of Naples Federico II, Naples, Italy; 3 Department of Translational Medical Sciences, University of Naples Federico II, Naples, Italy; 4 Department of Physics, Informatics and Mathematics, University of Modena and Reggio Emilia, Modena, Italy; 5 PhD School of Science, University of Basilicata, Potenza, Italy; 6 Division of Research and Development, Sanypet SpA, Padova, Italy; 7 Department of Veterinary Medicine and Animal Productions, University of Naples Federico II, Naples, Italy; 8 National Research Council (CNR), Nanoscience Istitute, Modena, Italy; 9 Department of Science, University of Basilicata, Potenza, Italy

**Keywords:** Immune pharmacology, Drug toxicity, Inflammatory response, DNA damage, Epigenetics

## Abstract

**Background:**

Oxytetracycline (OTC), which is largely employed in zootechnical and veterinary practices to ensure wellness of farmed animals, is partially absorbed within the gastrointestinal tract depositing in several tissues. Therefore, the potential OTC toxicity is relevant when considering the putative risk derived by the entry and accumulation of such drug in human and pet food chain supply. Despite scientific literature highlights several OTC-dependent toxic effects on human and animal health, the molecular mechanisms of such toxicity are still poorly understood.

**Methods:**

Here, we evaluated DNA damages and epigenetic alterations by quantitative reverse transcription polymerase chain reaction, quantitative polymerase chain reaction, chromatin immuno-precipitation and Western blot analysis.

**Results:**

We observed that human peripheral blood mononuclear cells (PBMCs) expressed DNA damage features (activation of ATM and p53, phosphorylation of H2AX and modifications of histone H3 methylation of lysine K4 in the chromatin) after the *in vitro* exposure to OTC. These changes are linked to a robust inflammatory response indicated by an increased expression of Interferon (IFN)-*γ* and type 1 superoxide dismutase (SOD1).

**Discussion:**

Our data reveal an unexpected biological *in vitro* activity of OTC able to modify DNA and chromatin in cultured human PBMC. In this regard, OTC presence in foods of animal origin could represent a potential risk for both the human and animal health.

## Introduction

The drug (4S,4aR,5S,5aR, 6S,12aS)-4-(dimethylamino)-3,5,6,10,12,12a-hexahydroxy-6-methyl-1,11-dioxo1,4,4a,5,5a,6,11,12a-octahydrotetracene-2-carboxamide, briefly oxytetracycline (OTC) is active towards a wide range of micro-organisms (Nelson & Levy, 2011), is efficiently absorbed in the duodenum forming complexes with metallic ions, is unstable at acid pH and its introduction along with food reduces its serum concentrations (Palmieri, Di Cerbo & Laurino, 2014). Moreover, such drug could accumulate within bone, skin, fat, tendons, muscles, liver and gastrointestinal tract (Agwuh & MacGowan, 2006).

OTC is commonly used in medicine and is one of the main antibiotics used in zootechnical and veterinary practices as feed supplement to ensure wellness of farmed animals (i.e., poultry, ovine, swine and livestock) (Graham et al., 2014; Brüning et al., 2014; Di Cerbo et al., 2014; Odore et al., 2015).

Several studies have investigated the potential toxicity of OTC ranging from teratogenic effects during pregnancy (Czeizel & Rockenbauer, 2000) to some effect on immune system (Glette et al., 1984; Potts et al., 1983; Van den Bogert & Kroon, 1982; Myers, Farrell & Henderson, 1995; Di Cerbo et al., 2016). Moreover, scientific literature suggested that the drug is able to inhibit or reduce catalase (Chi, Liu & Zhang, 2010) and affects avian cartilage degradation (Peters et al., 2002).

We recently demonstrated that OTC: (a) induces an *in vitro* inflammatory response characterized by T and non-T lymphocytes activation and Interferon (IFN)-*γ* release (Di Cerbo et al., 2016); (b) triggers the apoptosis of human and dog haematopoietic cells (Di Cerbo et al., 2016; Odore et al., 2015).

The potential OTC toxicity becomes more relevant when considering the potential risk derived by the eventuality of entry and accumulation of such drug in human and pet food with possible consequences on health (Palmieri, Di Cerbo & Laurino, 2014). In this regard, animal muscle, bone and fat are known to be the elective deposit for several antibiotics (Palmieri, Di Cerbo & Laurino, 2014; Macy & Poon, 2009) and are routinely employed for human and pet food production (Palmieri, Di Cerbo & Laurino, 2014).

In the light of the widespread use of OTC and considering the putative risk derived by the eventuality of entry and accumulation of such drug in human and pet food chain supply (Graham et al., 2014; Brüning et al., 2014; Di Cerbo et al., 2014; Nelson & Levy, 2011; Palmieri, Di Cerbo & Laurino, 2014), it is possible to speculate that the OTC accumulates in these edible tissues and that this occurrence represents the contact between the drug and the humans or companion animals (dogs and cats).

Here, we addressed the study over the relevance of some molecular mechanisms of drug toxicity and, specifically, on the genotoxic effect and epigenetic modifications potentially induced by OTC. This could be relevant since many of the effects observed could affect the gene expression and represent a potential risk for human and animal health.

### Materials & Methods

### Cells and incubation

Peripheral blood mononuclear cells (PBMCs) were obtained, as previously described (Di Cerbo et al., 2016). Briefly, we performed the centrifugation on Ficoll-Paque cushion (GE Healthcare, Uppsala Sweden) gradients of buffy coats obtained from six volunteer healthy donors. In order to inform the blood donors concerning the possibility to use minimal amount of their blood donation for scientific purpose, written informed consent (model n. 5526 of Azienda Ospedaliera Universitaria “FEDERICO II”, Naples, Italy) was obtained from each donor at the time of venous peripheral blood donation performed at Blood Trasfusional Center of Azienda Ospedaliera Universitaria “FEDERICO II”, Naples Italy, as established by Italian Law. All the experiments were performed anonymously, without any donor biographical reference. White blood cells have never been used to create a genome database.

To test the *in vitro* potential biochemical toxic role of OTC (Liquid Oxytetracycline 20% R, TreI, Reggio Emilia, Italy), the PBMCs (2.5 × 10^6^/ml) were incubated in presence of RPMI 1,640 medium with 10% FCS (Invitrogen, Carlsbad, CA, USA) alone or with 2 µg/ml OTC (Odore et al., 2015; Di Cerbo et al., 2016) at 37 °C for different times (6 h, 12 h, 24 h).

#### RNA extraction and qRT-PCR and qPCR

Total RNA was extracted using TRI Reagent (T9424, Sigma-Aldrich, St Louis, MO, USA). cDNA was synthesized in a 20 µl reaction volume containing 1 µg of total RNA, in accordance to the life technology protocol (High-Capacity cDNA Reverse Transcription Kit 4368814; Applied Biosystem, Thermofisher Scientific, Foster City, CA, USA). The products were stored at −20 °C until use. Quantitative reverse transcription polymerase chain reaction (qRT-PCR) and quantitative polymerase chain reaction (qPCR) were performed three times in six replicates on a 7,500 Real Time PCR System (Applied Biosystems) using the SYBR Green-detection system (SYBR select Master Mix, 4473369, Applied Biosystem). The following primers were used: IFN-*γ* mRNA, 5′-TGGAAAGAGGAGAGTGACAGA-3′ and 5′-CTGTTTTAGCTGCTGGCGAC-3′; type 1 superoxide dismutase (SOD1) mRNA 5′-CTAGCGAGTTATGGCGACGA-3′ and 5′-GTCTCCAACATGCCTCTCTTCA-3′; 18S, 5′-GCGCTACACTGACTGGCTC-3′ and 5′-CATCCAATCGGTAGTAGCGAC-3′.

### Chromatin Immuno-Precipitation (ChIP)

Cells were treated as indicated in *Cells and incubation* paragraph. The cells (∼2.5 × 10^6^ for each antibody) were crosslinked with a 1% formaldeyhyde/PBS solution for 10 min at room temperature, the reaction was stopped by the addition of glycine to a final concentration of 125 mM. Fixed cells were harvested and the pellet was resuspended in 1 ml of Lysis Buffer (10 mM Tris-HCl pH 8.0, 10 mM NaCl, 0.2% NP40) containing 1× protease inhibitor cocktail (Roche Applied Science, Basel, Switzerland). The lysates were sonicated in order to have DNA fragments from 300 to 600 bp. An aliquot (1/10) of sheared chromatin was used as input DNA. Sonicated samples were processed according to the manufacturer’s protocol of ChIP assay kit (Merck Millipore, Billerica, MA, USA). Samples were subjected to qPCR using the following primers: IFN-*γ* Promoter, 5′-GAA CAATGTGCTGCACCTCC-3′ and 5′-CACAGGTGGGCATAATGGGT-3′; SOD1 Promoter, 5′-CATCATTTTGCCAATTTCGCGT-3′ and 5′-CGAGTGGCCGGGAATGACT-3′.

Real Time-qPCRs were performed using the SYBR Green-detection system (SYBR select Master Mix, 4473369; Applied Biosystem).

#### Western blot preparation and analysis

Aliquots of the cells collected for ChIP were used for western blot. Cells were washed twice with cold phosphate-buffered saline (PBS) and nuclei were extracted using 1 ml of Lysis Buffer (10 mM Tris-HCl pH 8.0, 10 mM NaCl, 0.2% NP40) containing 1× protease inhibitor cocktail. Nuclear lysates were obtained accordingly with Nuclear Fractionation Protocol (Abcam, Cambridge, UK). *γ*H2AX was detected using part of the sonicated samples collected for ChIP. Lysates were cleared by centrifugation (13,000 rpm for 20 min). Protein concentrations were measured by Bio-Rad Protein Assay Dye Reagent Concentrate #500-0006. Equal amounts of cell extracts were then resolved by SDS–PAGE, transferred to nitrocellulose membranes, and immunoblotted using specific antibodies. Blots were detected using an ECL system (Lumilight Western Blotting Substrate, 12015200001, Roche).

### Antibodies

Anti-DNMT1 ab87656 (Abcam, Cambridge, UK), -H3K4me2 ab32356 (Abcam), -H3K4me3 ab1012 (Abcam),—Total H3 ab1791 (Abcam), -Menin sc-0200 (Santa Cruz Biotechnology); phosphoATM ab81292 (Abcam), -phospho-H2AX (07164, Merck Millipore), MCM7 sc-9966 (Santa Cruz Biotechnology), Normal rabbit IgG sc-2027 (Santa Cruz Biotechnology), Normal mouse IgG sc-2025 (Santa Cruz Biotechnology) and -p53 ab1101 (Abcam).

### Statistical analysis

Statistical significance between groups was determined using Student’s *t* test.

## Results and Discussion

### IFN-*γ* and SOD1 gene expression

**Figure 1:**
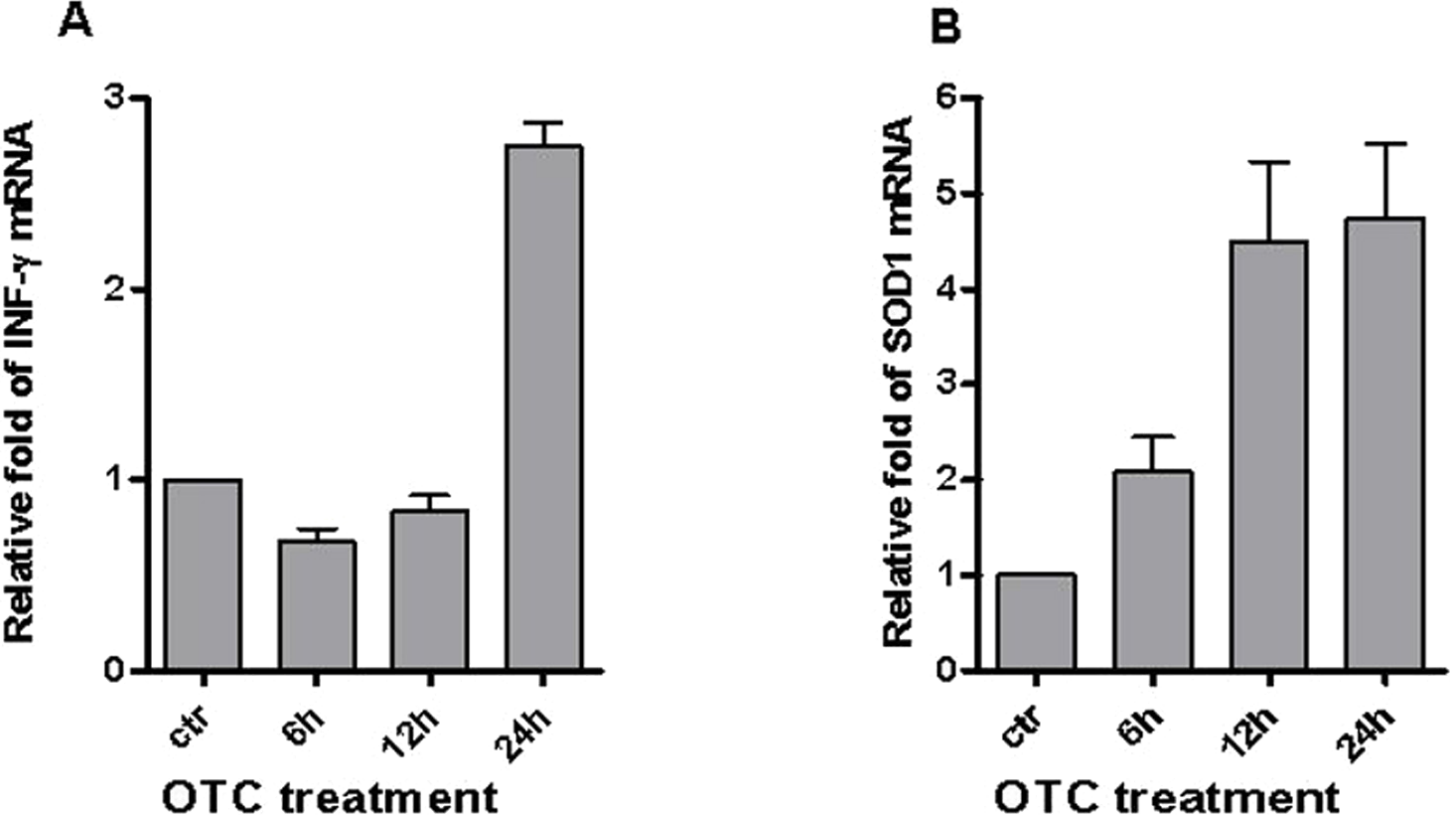
OTC induces IFN-*γ* and SOD1 mRNA. OTC significantly induces the increment of both IFN-*γ* and SOD1 mRNA. Total RNA was prepared from PBMC stimulated with OTC for 6, 12, 24 h, as indicated in ‘Materials & Methods’, and analyzed by qPCR with specific primers to IFN-*γ* (A) and SOD1 (B) mRNA normalized to 18S RNA levels. The statistical analysis derived from 2 experiments in triplicate (*n* ≥ 6; Mean ± SD).

We recently demonstrated the pro-inflammatory effect of OTC in causing both the IFN-*γ* secretion in T and non-T lymphocytes (Di Cerbo et al., 2016). Here, we evaluated the effect of drug treatment in the up-regulation of IFN-*γ* gene expression. Figure 1A shows that mRNA levels of IFN-*γ* robustly increased in PBMCs after 24 h of OTC incubation.

To investigate if OTC-mediated inflammatory condition could depend on oxidative stress, we evaluated whether the Cu–Zn Super Oxide Dismutase 1 (SOD1) could be increased after drug exposure. It is of note that one of the SOD1 is involved not only in oxidative metabolism but also in the T lymphocyte activation dependent on the accumulation of reactive oxygen species (Terrazzano et al., 2014). Our data (Fig. 1B) show that the mRNA levels of SOD1 increased from 12 to 24 h of OTC treatment.

The data reported in Fig. 1 showed that the enhancement of mRNA levels of IFN-*γ* occurred after 24 h of OTC-treatment, whereas the induction of SOD1 mRNA appeared already in 12 h. A possible explanation might be that SOD1 is a housekeeping gene (Minc et al., 1999) and its basal expression is usually higher than IFN-*γ* gene. Since the used *in vitro* model is based on freshly isolated PBMCs that are usually resistant to natural occurring apoptosis (Miyawaki et al., 1992), the increased SOD1 level after the drug incubation could be likely associated to the hypothesis of apoptosis induction upon chromatin and DNA damages (Norbury & Zhivotovsky, 2004; Barbosa et al., 2010).

These results suggest that the drug may affect some important cellular responses as the induction of a gene expression fostering the activation of previously observed immune response by T and non-T lymphocytes after *in vitro* OTC exposure (Di Cerbo et al., 2016).

### OTC generates genotoxic damage

Since the OTC is able to induce apoptosis (Odore et al., 2015; Di Cerbo et al., 2016), we investigated on the potential ability of such drug in causing DNA damage and, in reason of that, in inducing apoptosis. In particular, we evaluated the presence of genotoxic markers after *in vitro* drug treatment of PBMCs. In this regard, it is worth noting the role for Ataxia Telangiectasia mutated protein (ATM) that is a serin/treonin kinase activated in response to the DNA double strand break to promote cell cycle arrest, DNA repair and, if necessary, the cell death by apoptosis (Canman & Lim, 1998; Lee & Paull, 2007).

**Figure 2:**
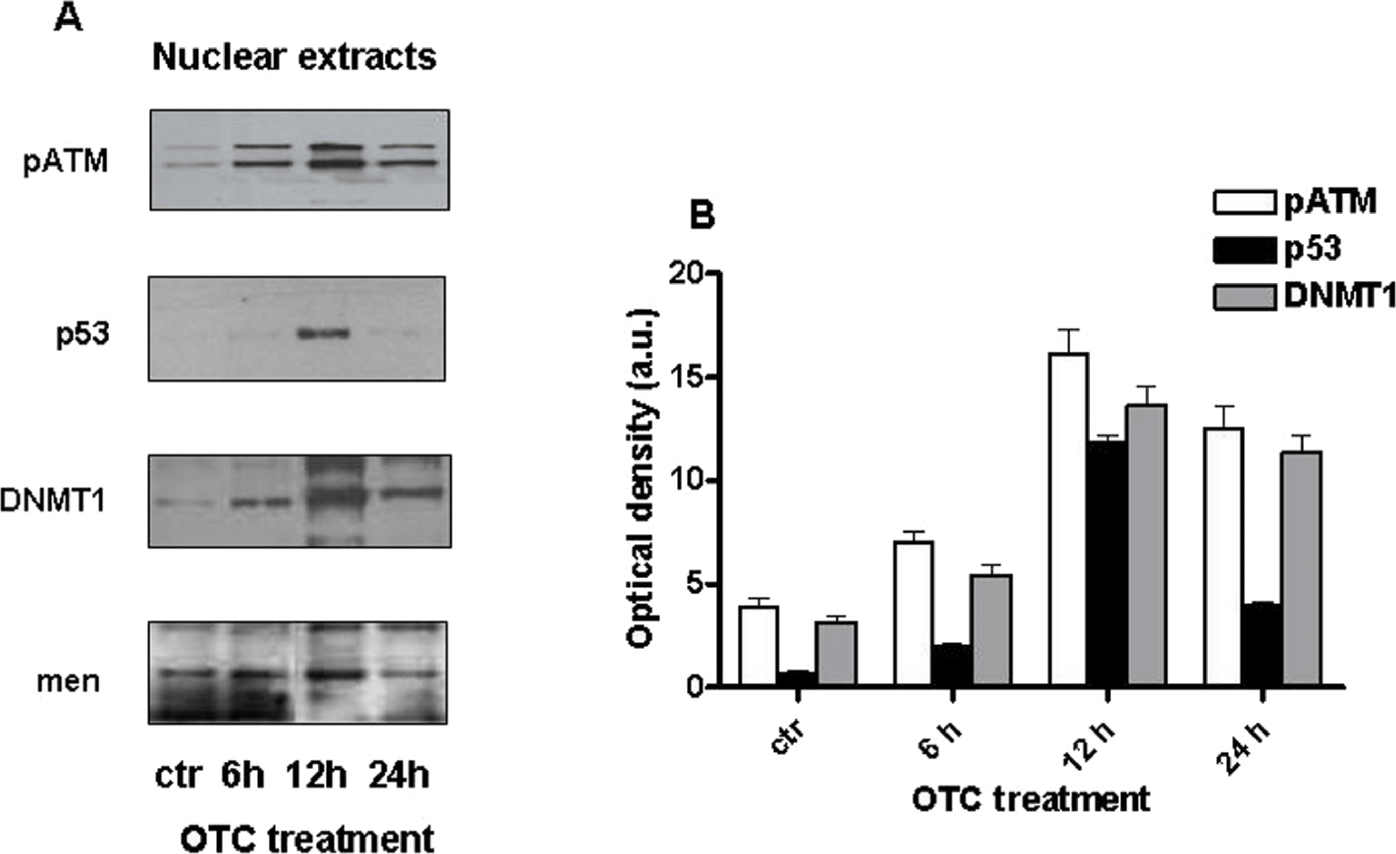
OTC induces genotoxic damage. Cells were treated with OTC for 6, 12 and 24 h and processed as indicated in ‘Material and Methods’. (A) the western blot for pATM, p53 and DNMT1 was performed using nuclear extract. Menin is reported as loading control; (B) quantification of the western blots normalized to Menin levels. Values are reported as Optical density (arbitrary units = a.u.).

As shown in Fig. 2A, the phosphorylated form of ATM (pATM) is clearly increased in human PBMCs after 6 h and 12 h of OTC incubation.

Furthermore, we investigated the levels of p53, as one of principal substrates of pATM and an important marker of DNA damage (Sakaguchi et al., 1998; Williams & Schumacher, 2016). Figure 2A indicates that p53 significantly increased after 12 h of drug exposure.

These observations suggest that some DNA damage may occur after OTC incubation.

One of the main epigenetic modifications involved in gene regulation is the DNA methylation (Hamidi, Singh & Chen, 2015). It is well known that DNA (cytosine-5)-methyltransferase 1 enzyme (DNMT1) is recruited to the chromatin, in response to the oxidative DNA damage, in order to inhibit gene transcription and to support DNA repair (Ding et al., 2016). It is of relevance that DNMT1 appears to increase after OTC treatment, following a similar kinetics of pATM and p53 (Fig. 2A).

Such evidence supports the idea that the enzymes could be recruited on the site of oxidative DNA damage occurred upon OTC incubation and cooperate each-other to induce chromatin modifications aimed to foster the DNA repair (Morano et al., 2014).

**Figure 3:**
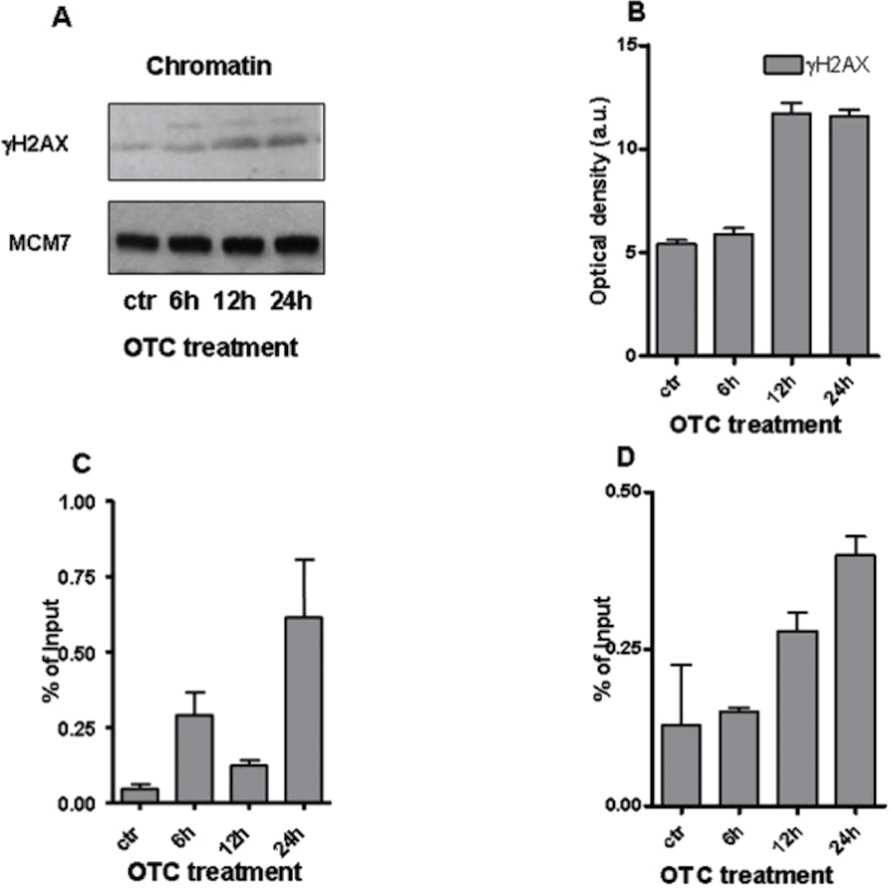
OTC and the chromatin changes. (A) The western blot for *γ*H2AX performed on chromatin extracts. MCM7 is reported as loading control; (B) Quantification of *γ*H2AX normalized to MCM7 levels. Values are reported as Optical density (arbitrary units = a.u.); qChIP analysis evideces that *γ*H2AX accumulates on IFN-*γ* (C) and SOD1 (D) promoters. Cells were treated with OTC as indicated, crosslinked and sonicated. The statistical analysis derived from at least 2 experiments in triplicate (*n* ≥ 6; Mean ± SD).

To better address the entity of DNA damage, we investigated the presence of DNA double strand break (DBS) markers. In particular, we evaluated the phosphorylated histone H2AX (*γ*H2AX) (Rogakou et al., 1998; Mah, El-Osta & Karagiannis, 2010) by performing western blot analysis on chromatin samples. Figures 3A and 3B shows the significant increase of *γ*H2AX with the highest peak from 12 to 24 h of drug treatment. Notably, the phosphorylated histone H2AX binds the regions of chromatin on the sites of DSB and DNA repair (Mah, El-Osta & Karagiannis, 2010). To better analyze the chromatin changes caused by the drug, we tested by ChIP assay the presence of *γ*H2AX on the promoters of genes of our interest. We observed the accumulation of *γ*H2AX at the site of promoter of IFN-*γ* gene (Fig. 3C). The presence of *γ*H2AX was also enhanced at the level of SOD1 gene promoter after 24 h of drug incubation (Fig. 3D).

Therefore, our results strongly suggest the correlation between DNA damage occurrence and OTC administration. Moreover, the increased levels of IFN-*γ* and SOD1 mRNAs (Fig. 1) appear to be linked to the function of *γ*H2AX, which cooperates with the induction of ATM mediated transcription (Singh et al., 2015).

## Epigenetic changes

Histone modification represents an epigenetic mechanism that affects gene transcription by altering the chromatin structure and DNA accessibility. Histone methylation can be associated with the different status of chromatin (Zhang, Cooper & Brockdorff, 2015). Here, we evaluated if OTC treatment could be correlated to alterations of histone methylation. More specifically, we investigated on the methylation status of lysine 4 of Histone 3 (H3K4) that is implicated in the regulation of gene activation (Barski et al., 2007; Ruthenburg, Allis & Wysocka, 2007).

**Figure 4:**
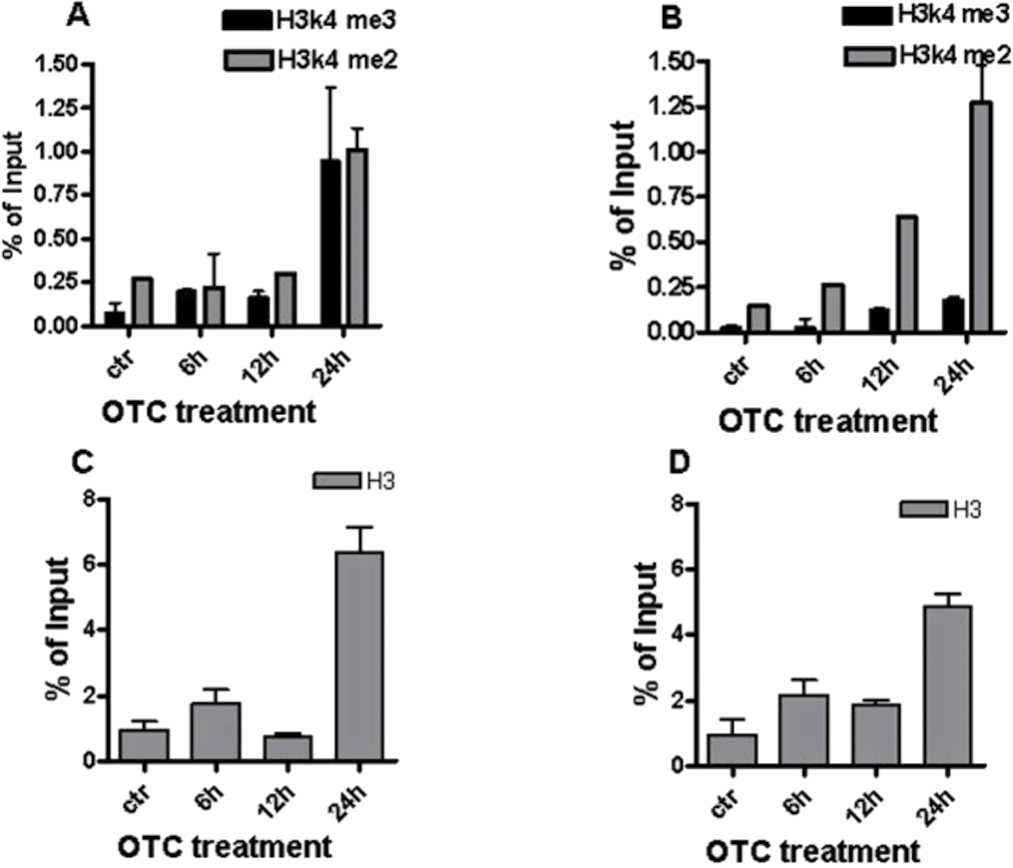
OTC and histone methylation. Methylation profile of histone H3K4 is induced by OTC on both the IFN-*γ* and SOD1 gene promoters. PMBC cells were exposed to OTC at the indicated times (0, 6, 12 and 24 h). qChIP was carried out using specific antibodies; (A) H3K4me3 and H3K4me2 occupancy at IFN-g promoter; (B): H3K4me2 and H3K4me3 occupancy at SOD1 promoter; (C) and (D); the TotalH3 occupancy at IFN-*γ* and SOD1 promoters respectively. The statistical analysis derived from at least 2 experiments in triplicate (*n* ≥ 6; Mean ± SD).

To this aim, we performed a ChIP for the promoter of the genes whose expression appeared to be modified by OTC. After 24 h of drug incubation, the increment of both the di-methylated (me2) and tri-methylated (me3) H3K4 is particularly evident for IFN-*γ* promoter (Fig. 4A), while the increase of H3K4 is more evident for the di-methylated form at the level of SOD1 promoter (Fig. 4B). These data are correlated with the observed activation of gene expression (Bernstein et al., 2005). Similar results are obtained from the analysis of total H3 histone levels (Figs. 4C and 4D).

Together, these data indicate that the OTC treatment can affect the status of chromatin.

## Conclusion

Despite scientific literature that has been suggesting the potential toxicity of OTC (Czeizel & Rockenbauer, 2000; Glette et al., 1984; Potts et al., 1983; Van den Bogert & Kroon, 1982; Myers, Farrell & Henderson, 1995), the mechanisms of the toxic effect of such drug is still poor understood.

We recently demonstrated that OTC induces *in vitro* inflammatory response (Di Cerbo et al., 2016) and apoptosis (Di Cerbo et al., 2016; Odore et al., 2015). Therefore, this and other suggestions open an interesting scenario on the toxicity of OTC that requires a greater understanding over the nature of observed toxic effects.

This study emphasized the toxicity of OTC, investigating over the molecular mechanisms involved in human PBMC inflammatory response. It is of note that the drug promoted a robust inflammatory response as represented by the increasing of IFN-*γ* mRNA levels. This result reflects and confirms the previously observed increment of IFN-*γ* production in T and non-T lymphocytes (Di Cerbo et al., 2016). In addition, OTC significantly induced SOD1 mRNA in the same experimental condition and cellular model. This evidence extends our previous observations on apoptosis induction after OTC exposure (Terrazzano et al., 2014; Odore et al., 2015).

In addition, we observed that OTC induces genotoxic damage as well as such drug recruits some enzymes implicated in the delicate balance between cell death and survival. Indeed, our data evidenced the increased levels of pATM, p53 and DNMT1 after drug incubation. It is of note that p53 is a substrate of pATM and is crucial to the cell cycle arrest and/or to induce the cell death by apoptosis (Norbury & Zhivotovsky, 2004), while DNMT1 represents a specific enzyme involved in some epigenetic changes (Hamidi, Singh & Chen, 2015), associated with the DNA damage (Rossetto et al., 2010).

Moreover, we observed the activation of *γ*H2AX, as a main DSB sensor protein, and suggested an epigenetic effect of OTC on the methylation status of H3K4 that is implicated in gene expression regulation (Barski et al., 2007; Ruthenburg, Allis & Wysocka, 2007). The increase of both me2 and me3 H3K4 occurred after OTC incubation and was evident for IFN-*γ* and SOD1 gene promoters.

Our data represent a preliminary step in the understanding of OTC toxicity, since the knowledge of the molecular mechanisms involved in the toxic effect may help in the generation of new drugs with reduced risk for human health.

In conclusion, it could be of great relevance to ascertain the possible acute and long term effects of OTC on human health. It is worth noting that the use of antibiotics for growth promotion is prohibited in Europe and it is considered a health hazard by WHO since 2006. The use of antibiotics in agriculture for non-therapeutic purposes is allowed in United States and Canada (FaAOF, 2014). Therefore, new regulations are urgently necessary to reduce antibiotic contaminants in foods as well as the antibiotic resistance phenomenon (US Government Publishing Office, 2014).

### Study Limitations

Notably, the current study incurs some limitations that are not addressable without further researches. In this regard, our study did not perform chemical-pharmaceutical and pharmacological test to evaluate the molecular complexity and/or stability of the used OTC or to verify the possible presence of active sub-products generated during *in vitro* tests. In addition, this study did not address any chemical evaluation of the excipients (i.e., fillers, binders, dyes, flavorings, preservatives and other materials) present in the here used commercial liquid formulation of OTC drug employed in veterinary medicine. Therefore, further evaluations are required to complete the significance of OTC toxicity. In particular, the absence of *in vivo* experiments, able to confirm the *in vitro* observed OTC toxicity, represents the main relevant limitation. Therefore, clinical studies are required to ascertain the effect of the drug in inducing the inflammatory status in animals and/or in humans.

##  Supplemental Information

10.7717/peerj.3236/supp-1 Supplemental Information 1 Raw data of Western blot related to Fig. 2A and Fig. 3A

## Additional Information and Declarations

### Data Availability

The following information was supplied regarding data availability: The raw data has been supplied as Data S1.
